# Use of matrix-assisted laser desorption/ionisation-time of flight mass spectrometry analyser in a diagnostic microbiology laboratory in a developing country

**DOI:** 10.4102/ajlm.v6i1.598

**Published:** 2017-12-08

**Authors:** Atang Bulane, Anwar Hoosen

**Affiliations:** 1 Department of Medical Microbiology & Virology, University of the Free State, Bloemfontein, South Africa

## Abstract

**Background:**

Rapid and accurate identification of pathogens is of utmost importance for management of patients. Current identification relies on conventional phenotypic methods which are time consuming. Matrix-assisted laser desorption/ionisation-time of flight mass spectrometry (MALDI-TOF MS) is based on proteomic profiling and allows for rapid identification of pathogens.

**Objective:**

We compared MALDI-TOF MS against two commercial systems, MicroScan Walkaway and VITEK 2 MS.

**Methods:**

Over a three-month period from July 2013 to September 2013, a total of 227 bacteria and yeasts were collected from an academic microbiology laboratory (*N* = 121; 87 Gram-negatives, seven Gram-positives, 27 yeasts) and other laboratories (*N* = 106; 35 Gram-negatives, 34 Gram-positives, 37 yeasts). Sixty-five positive blood cultures were initially processed with Bruker Sepsityper kit for direct identification.

**Results:**

From the 65 blood culture bottles, four grew more than one bacterial pathogen and MALDI-TOF MS identified only one isolate. The blood cultures yielded 21 Gram-negatives, 43 Gram-positives and one *Candida*. There were 21 *Escherirchia coli* isolates which were reported by the MALDI-TOF MS as *E. coli*/*Shigella*. Of the total 292 isolates, discrepant results were found for one bacterial and three yeast isolates. Discrepant results were resolved by testing with the API system with MALDI-TOF MS showing 100% correlation.

**Conclusion:**

The MALDI-TOF MS proved to be very useful for rapid and reliable identification of bacteria and yeasts directly from blood cultures and after culture of other specimens. The difference in time to identification was significant for all isolates. However, for positive blood cultures with minimal sample preparation time there was a massive difference in turn-around time with great appreciation by clinicians.

## Introduction

In clinical microbiology laboratories, the identification of microorganisms in patient specimens has historically been based on the detection of pathogen-specific phenotypic characteristics.^1^ These include microscopic and colony morphology features and biochemical phenotypes that can be detected with either manual or automated methods.^1^ The most commonly used automated systems in South Africa are the VITEK 2 MS and MicroScan Walkaway systems. Although, these systems allow for the identification of most bacterial isolates with great accuracy, they are costly and time consuming. They rely on the active metabolic processes of the pathogen and as a result long incubation periods are required. The introduction of the matrix-assisted laser desorption/ionisation-time of flight mass spectrometry (MALDI-TOF MS) method, which is based on proteomic profiling, has provided fast, reliable and cost-effective identification of bacteria and yeast.^2,3,4^ The MALDI-TOF MS assay has also shown the ability to directly identify bacteria or fungi from positive blood cultures.^5,6,7^ This greatly reduces the turn-around time for patients with suspected sepsis. At present, blood culture analysis takes at least 24 h or more before a definitive diagnosis is achieved.^8^

MALDI-TOF MS has been routinely used in clinical laboratories in European countries from 2009 but is not widely used in South Africa. Several reports show that it is reproducible, and produces results that are comparable to genome sequencing.^9,10,11^ In this study, the MALDI-TOF MS was evaluated for diagnostic microbiology in a developing country. The first part of the study compared MALDI-TOF MS identification results with identification by the MicroScan Walkaway system (Siemens Healthcare Diagnostics, Sacramento, California, United States) at an academic laboratory. The second part of study compared isolates from a regional hospital and two private pathology laboratories that used the VITEK 2 MS (bioMérieux, Marcy l’Etoile, France). The last part of study compared organisms from blood culture vials when a positive signal was emitted from the BacT/Alert 3D system (bioMérieux, Marcy l’Etoile, France).

## Methods

### Collection and analysis of clinical isolates

Samples were collected from 15 July 2013 to 30 September 2013 (three-month period). Bacteria and yeast isolates retrieved from clinical samples from an academic diagnostic microbiology laboratory (National Health Laboratory Service [NHLS], Universitas Hospital, Bloemfontein, Free State, South Africa) and three other laboratories, one at Kimberly Hospital (Northern Cape, South Africa) and two private pathology laboratories (Bloemfontein, Free State, South Africa), were analysed. The laboratories identified isolates to the species level with standard biochemical methods using either the MicroScan Walkaway system (Siemens Healthcare Diagnostics, Sacramento, California, United States) or the VITEK 2 MS system (bioMérieux, Marcy l’Etoile, France). Isolates were tested in parallel with the MALDI-TOF MS assay (Bruker Daltonics, Bremen, Germany). The technician who carried out the MALDI-TOF MS assay was blinded to the identity of the isolates. The analysis of the isolates from the academic diagnostic microbiology laboratory was done in real time, whereas the samples from regional and private laboratories were assayed in batches.

### Blood culture analysis

This analysis was only performed for the samples from the academic diagnostic microbiology laboratory. Only the initial positive culture from each patient was used to avoid duplicate analyses of samples from the same septic episode. Charcoal-free, positive blood culture bottles were Gram-stained and protein was extracted according to manufacturer’s instructions using the Bruker Sepsityper protein extraction kit (Bruker Daltonics, Bremen, Germany). In brief, 200 μl of lysis buffer was added to 1.0-ml aliquot of positive blood culture (BacT/Alert 3D MS aerobic, anaerobic bottles). This mixture was centrifuged for 1 min at 13 000 rpm. The supernatant was removed and the pellet was re‑suspended in 1 ml of washing buffer. The supernatant was discarded and the pellet was further re-suspended in 75% ethanol. Ethanol-formic acid extraction was then performed as per the manufacturer’s instructions. After extraction 1 μl of protein supernatant was spotted on a 96-spot MALDI-TOF MS target plate, overlaid with matrix and analysed using Biotyper version 3.0 according to the manufacturer’s instructions. The only modification to this procedure was that instead of a 1.0 ml aliquot, a 3 ml aliquot was used for protein extraction from positive paediatric blood culture bottles. The *Escherirchia coli* ATCC 25922 reference strain was used as a positive control, and matrix with no organism was used as a negative control in the analysis.

### Matrix-assisted laser desorption/ionisation-time of flight mass spectrometry analysis

Clinical isolate identification by the MALDI-TOF MS assay was performed according to the manufacturer’s instructions. In brief, the isolated colonies were directly applied onto steel MSP 96 MALDI ground steel target plates (Bruker Daltonics, Bremen, Germany). The yeast isolates were overlaid with 2 μl formic acid for protein extraction and 2 μl of alpha-cyano-4-hydroxy cinamic acid matrix, while the bacterial isolates were directly overlaid with 2 μl alpha-cyano-4-hydroxy cinamic acid matrix. The Microflex LT instrument was calibrated twice a week by using the Bruker Daltonics bacterial test standard. When scores greater than 2.0 were generated, they were classified as ‘high-confidence’ (secure species), whereas scores between 1.7 and 1.99 were classified as ‘intermediate confidence’ (genus only) and scores of less than 1.7 were classified as ‘unacceptable’. The results obtained by MALDI-TOF MS were compared to the results obtained by the conventional methods using the MicroScan Walkaway system and VITEK 2 MS after analysis by the respective laboratories. The API Coryne V 2.0 (bioMérieux, Marcy l’Etoile, France) was used to resolve discrepancies between MALDI-TOF MS and Vitek 2 MS results.

The isolated colonies were directly applied onto steel MSP 96 MALDI ground steel target plates (Bruker Daltonics, Bremen, Germany). The yeast isolates were overlaid with 2 μl formic acid for protein extraction and 2 μl of alpha-cyano-4-hydroxy cinamic acid matrix, while the bacterial isolates were directly overlaid with 2 μl alpha-cyano-4-hydroxy cinamic acid matrix. The plates were analysed by the MALDI-TOF MS machine.

### Turn-around time for identification of blood culture isolates

The turn-around time for identification from blood cultures was determined by calculating the time elapsed between the incubation of the sample in the automated instrument and identification by standard laboratory methods. The MALDI-TOF MS assay turn-around time was calculated from the instrument flagging a positive blood culture to the time when the MALDI-TOF MS machine completed the interpretation of the spectra.

## Results

A total of 227 isolates were evaluated over the three-month study period (academic diagnostic microbiology laboratory *N* = 121; other laboratories *N* = 106). Of the 227 isolates, 122 were Gram-negative bacteria, 41 Gram-positive bacteria and 64 yeast isolates. Among the isolates from the academic diagnostic microbiology laboratory, 87 were Gram-negative, seven Gram-positive and 27 yeast isolates, whereas 35 Gram-negatives, 34 Gram-positives and 37 yeast isolates were from the other laboratories. The 122 Gram-negative isolates analysed by MALDI-TOF MS were 100% concordant at the species level with the VITEK 2 MS and MicroScan Walkaway systems (Table 1). A total of 98 (80.3%) isolates generated a high confidence score, 22 (18.0%) had an intermediate score and two (1.6%) had a very low confidence score (*Proteus mirabilis* and *Hafnia alvei*). Despite their low score, identification by both the MicroScan Walkaway system and the VITEK 2 MS system was in concordance with the MALDI-TOF MS identification.

**TABLE 1:** Gram-negative bacterial isolates identified (*N* = 122), Bloemfontein, South Africa, 15 July 2013 to 30 September 2013.

Organisms identified by MALDI-TOF MS	No. of isolates identified on other platforms		Total no. of isolates analysed	Isolates identified by MALDI-TOF MS by score No. (%)		
	MicroScan Walkway system†	VITEK 2 MS‡		< 1.7	1.7–1.99	> 2.0
*P. mirabilis*	17	5	22	1	3	18
*K. pneumoniae*	16	6	22	0	5	17
*E. coli*	14	5	19	0	3	16
*Steno maltophilia*	6	2	8	0	3	5
*P. aeruginosa*	10	5	15	0	0	15
*A. baumanii*	14	0	14	0	1	13
*S. marcenscences*	5	0	5	0	3	2
*E. aerogenes*	2	0	2	0	0	2
*Delftia acidovorans*	1	0	1	0	1	0
*E. cloacae*	1	1	2	0	0	2
*M. morganii*	1	2	3	0	1	2
*H. influenzae*	0	1	1	0	0	1
*Chry. indologenes*	0	3	3	0	2	1
*H. alvei*	0	1	1	1	0	0
*Y. enterocolitis*	0	1	1	0	0	1
*Salmonella spp*	0	3	3	0	0	3
**Total**	87	35	122	2 (1.6%)	22 (18.0%)	98 (80.3%)

† Platform used at academic diagnostic microbiology laboratory, Universitas Hospital, Bloemfontein, South Africa.

‡ Platform used at three other laboratories, one at Kimberly Hospital (Northern Cape, South Africa) and two private pathology laboratories (Bloemfontein, South Africa).

For the 41 Gram-positive isolates identified by MALDI-TOF MS (Table 2), 26 (63.4%) generated a high confidence score, 14 (34.1%) had an intermediate score and 1 (2.4%) had a poor score. Forty out of the 41 Gram-positive isolates showed concordance at the species level while one was discrepant. Of the 40 cpncordant isolates, seven were from the MicroScan Walkaway system and 33 from the VITEK 2 MS system. Despite the poor score for the *Streptococcus pneumoniae* isolate, there was concordance with the VITEK 2 MS. The discrepant isolate was identified as *Arthrobacter spp* by MALDI-TOF MS with an intermediate score value (1.7–1.9), but was identified by the VITEK 2 MS as *Staphlococcus aureus*. However, the isolate was confirmed as *Arthrobacter spp* by the API Coryne (bioMérieux, Marcy l’Etoile, France) with a score value of 64.5%.

**TABLE 2:** Gram-positive bacterial isolates identified (*N* = 41), Bloemfontein, South Africa, 15 July 2013 to 30 September 2013.

Organisms identified by MALDI-TOF MS	No. of isolates identified on other platforms		Total no. of isolates analysed	Isolates identified by MALDI-TOF MS by score No. (%)		
	MicroScan Walkway system†	VITEK2 MS‡		< 1.7	1.7–1.99	> 2.0
*Staph. epidermidis*	3	4	7	0	3	4
*Enterococcus faecalis*	2	6	8	0	3	5
*Enterococcus faecium*	2	4	6	0	2	4
*Strep. pneumoniae*	0	3	3	1	0	2
*Staph. aureus*	0	10	10	0	3	7
*Staph. saprophyticus*	0	1	1	0	1	0
*S. agalactiae*	0	1	1	0	0	1
*M. luteus*	0	1	1	0	0	1
*S. pyogenes*	0	2	2	0	1	1
*Arthrobacter spp*	0	1	1	0	1	0
*C. perfringes*	0	1	1	0	0	1
**Total**	7	34	41	1 (2.4%)	14 (34.1%)	26 (63.4%)

† Platform used at academic diagnostic microbiology laboratory, Universitas Hospital, Bloemfontein, South Africa.

‡ Platform used at three other laboratories, one at Kimberly Hospital (Northern Cape, South Africa) and two private pathology laboratories (Bloemfontein, South Africa).

Among the 64 yeast isolates analysed by MALDI-TOF MS (Table 3), 48 (75.0%) showed an acceptable identification score of 1.7 or higher. Of the 64 isolates, 16 (25.0%) generated a high score, 32 (50.0%) had an intermediate score and 16 (25.0%) had a poor identification score. For the 16 yeast isolates with a poor identification score by MALDI-TOF MS, their identities were found to be in agreement with the MicroScan Walkaway system for the isolates from academic laboratory and VITEK 2 MS for the isolates from other laboratories. Among the isolates with an intermediate score, two (4.2%) isolates (1.7–1.99) were identified as *Candida parapsilosis* and *Candida dubliniensis* by MALDI-TOF MS but were identified as *Candida albicans* by the MicroScan Walkaway. However, VITEK 2 MS also identified them as *C. parapsilosis* and *C. dubliniensis*.

**TABLE 3:** Yeast isolates identified by MALDI-TOF MS (*N* = 64), Bloemfontein, South Africa, 15 July 2013 to 30 September 2013.

Organisms identified by MALDI-TOF MS	No. of isolates identified on other platforms		Total no. of isolates analysed	Isolates identified by MALDI-TOF MS by score No. (%)		
	MicroScanMS†	VITEK2 MS‡		< 1.7	1.7–1.99	> 2.0
*C. albicans*	14	28	42	15	18	9
*C. parapsilosis*	10	1	11	0	8	3
*C. glabrata*	2	5	7	0	3	4
*C. tropicalis*	0	2	2	0	2	0
*C. dubliensis*	1	1	2	1	1	0
Total	27	37	64	16 (25.0%)	32 (50.0%)	16 (25.0%)

† Platform used at academic diagnostic microbiology laboratory, Universitas Hospital, Bloemfontein, South Africa.

‡ Platform used at three other laboratories, one at Kimberly Hospital (Northern Cape, South Africa) and two private pathology laboratories (Bloemfontein, South Africa).

There was 100% concordance for bacterial identification between the MALDI-TOF MS assay and the blood cultures (Tables 4 and 5). In total, 65 blood culture bottles were first Gram stained and then analysed by MALDI-TOF MS; 60 (92.0%) were monomicrobial, four (6.0%) were polymicrobial and one (2.0%) was cultured a yeast. Ultimately 21 (32.0%) blood culture isolates were classified as Gram-negative bacteria, 43 (66.0%) as Gram-positive bacteria and 1 (2.0%) as *Candida spp*.

**TABLE 4:** Gram-negative bacteria identified directly from blood cultures by MALDI-TOF MS (*N* = 21), Bloemfontein, South Africa, 15 July 2013 to 30 September 2013.

Organisms identified by MALDI-TOF MS	No. of isolates from MicroScan MS†	MALDI-TOF MS score		
		< 1.7	1.7–1.99	> 2.0
*P. aeruginosa*	4	0	2	2
*K. pnuemoniae*	7	0	3	4
*A. baumanii*	5	0	1	4
*E. cloacae*	1	0	0	1
*P. acnes*	1	0	1	0
*E. coli*	2	0	0	2
*C. koseri*	1	0	0	1
**Total**	21	0	7 (33%)	14 (67%)

† Platform used at academic diagnostic microbiology laboratory, Universitas Hospital, Bloemfontein, South Africa.

**TABLE 5:** Gram-positive bacteria identified directly from blood cultures by MALDI-TOF MS (*N* = 43), Bloemfontein, South Africa, 15 July 2013 to 30 September 2013.

Organisms identified by MALDI-TOF MS	No. of isolates from MicroScan MS†	MALDI-TOF MS score		
		< 1.7	1.7–1.99	> 2.0
*Staph. epidermidis*	26	3	10	13
*Staph. hominis*	3	1	0	2
*Micrococcus luteus*	2	0	0	2
*E. faecalis*	5	0	1	4
*E. faecium*	6	0	0	6
*E. avium*	1	0	1	0
**Total**	43	4 (9%)	12 (28%)	27 (63%)

† Platform used at academic diagnostic microbiology laboratory, Universitas Hospital, Bloemfontein, South Africa.

Of the four polymicrobial blood bottles, the MALDI-TOF MS assay correctly identified only one organism (Table 6). *Candida spp* (score 1.84) was identified as the *Candida guiliermondii* (ana) by the MALDI-TOF MS, while the MicroScan Walkaway system identified it as *Candida zeylanoides*. The yeast was further tested by the VITEK 2 MS and was identified as *C. guiliermondii.*

**TABLE 6:** Four polymicrobial blood cultures and direct identification by MALDI-TOF MS (*N* = 4), Bloemfontein, South Africa, 15 July 2013 to 30 September 2013.

Conventional laboratory identification	Direct MALDI-TOF MS identification
*A. baumanii* & *E. faecium*	*E. faecium*
CNS & *A. baumanii*	*S. epidermidis*
*A. baumanii* & *Enterococcus spp*	*E. avium*
CNS & *C. koseri*	*C. koseri*

CNS, coagulase negative staphylococci

The average identification time by MALDI-TOF MS for 65 positive blood culture bottles from the time a signal was generated by the blood culture machine was 35 min. The conventional methods required 48 h.

## Discussion

Despite the common use of MALDI-TOF MS in laboratories based in developed countries, this system is not frequently used in developing countries. For this reason, few data are available on the efficacy of MALDI-TOF MS for routine diagnosis of bacterial and yeast identification in microbiology laboratories in developing countries such as South Africa. In this study, the utility of MALDI-TOF MS for the identification of bacterial and yeast species either from clinical specimens or directly from blood culture broth was evaluated and compared with routinely used accepted methods (MicroScan Walkaway system and VITEK 2 MS) in a resource-limited country.

Current procedures, such as conventional and automated methods that are used in blood culture identification, delay pathogen identification for hours, or even days when fastidious bacteria are involved. In this study, an average of 48 h was required for pathogen identification from blood cultures, whereas it took a mere 35 min on average for extraction and identification of both bacteria and yeast directly from the same blood cultures by the MALDI-TOF MS. This system has shown the potential to reduce delays that currently exist between blood culture sampling and the availability of the results to clinicians and hence allows initiation of early species- or genus-oriented empirical treatment.

The rapid identification of the causative agent is not only important in treatment selection; according to other studies based on rapid identification techniques, it also reduces therapeutic costs.^12^ Although it has been reported that the reliability for MALDI-TOF MS in identification of Gram-positive bacteria is lower when compared to Gram-negative bacteria in blood cultures,^12^ in the current study MALDI-TOF MS was able to correctly identify all Gram-positive isolates that were supplied and the majority had good score values.

Polymicrobial blood cultures were found in 4 of the 65 (6.2%) positive samples, and MALDI-TOF MS could only identify 1 of the pathogens among these mixed blood cultures. The identification score values ranged between 1.45 and 2. This is a limitation for the use of the MALDI-TOF MS system for identification of pathogens directly from polymicrobial blood samples.

One *Candida spp* was identified directly from a positive blood culture sample. The *Candida spp* was identified as *C. guiliermondii* (ana) by the MALDI-TOF MS with a score value of 1.84, whereas the MicroScan Walkway system identified it as *C. zeylanoides*. The yeast isolate was further tested by the VITEK 2 MS and identified as *C. guiliermondii*.

When MALDI-TOF MS was evaluated for the identification of bacterial isolates, results showed that MALDI-TOF MS allows excellent identification at the species level for all the Gram-positive and Gram-negative bacteria, as a significant proportion of bacterial isolates identified showed high correlation with the MicroScan Walkaway and VITEK 2 MS results (99.4%). According to literature on the use of MALDI-TOF MS, species identification can be attained at a threshold of 2.0.^13^ However, among the 292 samples analysed in the current study, 107 had an identification score of less than 2 by MALDI-TOF MS. Interestingly, the MALDI-TOF MS identities of these 107 isolates correlated with those given by MicroScan Walkaway system and VITEK 2 MS.

Similar results have also been reported in other studies where a score value lower than 2 still resulted in reliable species identification.^3,14,15,16^ It was hypothesised that the low identification score by MALDI-TOF MS may be due to the low analyte concentration rather than a low degree of relatedness to the information in the database. The inclusion of low-scoring samples in the current study increased the sensitivity of the MALDI-TOF MS. Bacterial isolates that were identified as *S. pneumoniae* by the MALDI-TOF MS were confirmed as such by MicroScan Walkaway system.

For identification of yeast isolates, MALDI-TOF MS was able to reliably identify 62 of the 64 (96.9%) isolates that were included in the current study. A total of four different yeast species were identified in correlation with the VITEK 2 MS and MicroScan Walkaway systems. There were two discrepant yeast isolates from a swab and catheter tip which were identified as *C. dubliniensis* (score 1.85) and *C. parapsilosis* (score 1.91) by MALDI-TOF MS. The two isolates were both identified as *C. albicans* by the MicroScan Walkaway system and the MALDI-TOF MS identification was confirmed by the VITEK 2 MS. The correct identification of the *Candida spp* is important, since the infection and clinical impact of other *Candida spp* other than *C. albicans* seem to be increasing among HIV-positive patients, which leads to challenges in empirical antifungal treatment.^19^ *Candida spp*, including *Candida glabrata* and *C. parapsilosis*, are known to be resistant to fluconazole,^20^ which is one of the drugs most widely used in the treatment of systemic fungal infections. Rapid identification of this species is essential for proper treatment and management.

### Limitations

The sample size for direct identification from blood cultures was relatively small and there was only one yeast isolate. A larger sample including more yeast isolates would have provided useful information.

### Conclusion

The MALDI-TOF MS assay proved to be very useful for rapid and reliable identification of bacterial and yeast pathogens directly from blood cultures and isolates from other specimens at academic and private pathology laboratories in a developing country. The difference in time to identification for all isolates was significant between the MALDI-TOF MS and the two other automated systems (MicroScan Walkaway and VITEK 2 MS). However, for positive blood cultures with their minimal sample preparation time, there was a massive difference in turn-around time, contributing to great laboratory efficiency.
